# *Helicobacter pylori* HP0135 Is a Small Lipoprotein That Has a Role in Outer Membrane Stability

**DOI:** 10.3390/molecules30020204

**Published:** 2025-01-07

**Authors:** Doreen Nguyen, Rachel G. Ivester, Kyle Rosinke, Timothy R. Hoover

**Affiliations:** 1 Department of Microbiology, University of Georgia, Athens, GA 30602, USA; doreen.nguyen25@uga.edu (D.N.); krosinke@uga.edu (K.R.); 2 Department of Microbiology and Cell Science, University of Florida, Gainesville, FL 32611, USA; rachel.ivester@ufl.edu

**Keywords:** small protein, lipoprotein, outer membrane, *Helicobacter pylori*

## Abstract

*Helicobacter pylori* is a Gram-negative bacterium and human pathogen that is linked to various gastric diseases, including peptic ulcer disease, chronic gastritis, and gastric cancer. The filament of the *H. pylori* flagellum is surrounded by a membranous sheath that is contiguous with the outer membrane. Proteomic analysis of isolated sheathed flagella from *H. pylori* B128 identified the lipoprotein HP0135 as a potential component of the flagellar sheath. HP0135 is a small protein, with the mature HP0135 lipoprotein only 28 amino acid residues in length. Deletion of *hp0135* in *H. pylori* B128 resulted in morphological abnormalities that included extensive formation of outer membrane vesicles and increased frequency of mini-cells. Introducing a plasmid-borne copy of *hp0135* into the *H. pylori* Δ*hp0135* mutant suppressed the morphological abnormalities. The phenotype of the Δ*hp0135* mutant suggests HP0135 has roles in stabilizing the cell envelope and cell division.

## 1. Introduction

*Helicobacter pylori*, a diderm (i.e., Gram-negative bacterium) that belongs to the phylum Campylobacterota, colonizes the human stomach of approximately half the population worldwide [1,2]. Colonization of the gastric epithelium by *H. pylori* can result in peptic ulcer disease and chronic gastritis and is also a major risk factor for gastric cancer and mucosa-associated lymphoid tissue lymphoma [3,4,5]. Many of the virulence factors that have been identified in *H. pylori* are outer membrane (OM) lipoproteins [6]. These lipoproteins have a wide range of physiological roles, including adherence to epithelial cells [7,8], virulence [9,10], migration within host cells [11], and competence [12].

The secretion, processing, and acylation of bacterial lipoproteins is a multistep process. The prolipoprotein contains an N-terminal signal peptide that usually directs the protein for secretion across the cell membrane by the Sec pathway, although some lipoproteins are transported by the Tat pathway [13,14]. The C-terminus of the lipoprotein signal peptide contains an invariant cysteine residue that is acylated and becomes the N-terminal residue of the protein upon cleavage of the signal peptide [13,14]. The thiol group of the conserved cysteine residue is acylated by a preprolipoprotein diacylglyceryl transferase, Lgt, following the transport of the prolipoprotein across the cell membrane [15]. The signal peptide is then cleaved by the lipoprotein signal peptidase, Lsp, placing the acylated cysteine at the N-terminus of the lipoprotein [16,17]. The N-terminal amine group of the cysteine is then acylated by an N-acyl transferase, Lnt, to form a triacylated lipoprotein [18].

Lipoproteins targeted for the OM are removed from the inner membrane and translocated across the periplasmic space by the localization of the lipoproteins (Lol) transport pathway [19]. The *Escherichia coli* Lol system, which represents the canonical pathway, consists of five proteins. The ATP-binding cassette (ABC) transporter LolCDE removes lipoproteins from the inner membrane and delivers them to the periplasmic chaperone LolA [20,21]. Lipoproteins bound to LolA are then transferred to the OM receptor LolB for insertion into the OM [20,22]. Many bacterial species, including *H. pylori*, either lack or use alternative components of the canonical Lol pathway. These deviations include the substitution of the LolCE proteins with LolF [23] and the absence of a LolB homolog [10,14]. The LolA protein of *Caulobacter vibrioides*, which lacks a LolB homolog, has both lipoprotein chaperone and membrane insertion activities [24].

Flagellum-mediated motility of *H. pylori* is required for host colonization of animal models [25,26]. The *H. pylori* flagellum is encased in a membranous sheath that is contiguous with the OM, a feature that is shared with many other *Helicobacter* and *Vibrio* species [27]. Examining the *H. pylori* flagellum and associated sheath, we identified the small lipoprotein HP0135, which is only 28 amino acid residues in length, as a component of the flagellar sheath proteome. Bacterial proteins of ≤50 amino acids, referred to as small proteins or microproteins, have been understudied as they are often overlooked in bioinformatic, biochemical, and genetic approaches. About 30% of all the microproteins identified in *E. coli* are either predicted or known to localize to the inner membrane [28]. Recent studies have demonstrated roles for membrane-associated small proteins in a range of cellular processes, including solute transport, signal transduction, respiration, stress response, sporulation, and cell division [28,29,30]. Deletion of *hp0135* in *H. pylori* B128 resulted in extensive formation of OM vesicles (OMVs), which are nanoparticles derived from the OM and are part of normal bacterial growth both in vitro and in vivo [31]. In addition to an abundance of OMVs, the Δ*hp0135* mutant displayed increased numbers of mini-cells, which are abnormally short cells that result from cell division occurring near the cell pole. As occurs in many other bacteria, the site of cell division in *H. pylori* is influenced by the Min system [32,33], which consists of the proteins MinC, MinD, and MinE and mediates the assembly of the FtsZ-ring at the midcell [34]. The morphological anomalies of the *H. pylori* Δ*hp0135* mutant were suppressed by introducing a plasmid-borne copy of *hp0135*, suggesting that loss of HP0135 was responsible for the aberrant morphology of the Δ*hp0135* mutant and that HP0135 has roles in cell envelope maintenance and cell division.

## 2. Results

### 2.1. HP0135 Is a Small Lipoprotein Associated with the H. pylori Flagellar Sheath

As part of a study to examine the protein content of the *H. pylori* flagellar sheath, flagella were sheared from *H. pylori* B128 cells, isolated with their accompanying sheaths, and analyzed by mass spectroscopy. A total of 206 non-ribosomal proteins with high confidence for protein false discovery rate (FDR) were identified in the flagellar preparation (Table S1). Proteins listed in Table S1 are ordered according to the area under the peaks of the peptide ions associated with the protein, which roughly correlates to the relative abundance of the protein. Consistent with their expected abundance in the flagellar preparation, the major flagellin (FlaA) had the highest area, and the minor flagellin (FlaB) had the fourth highest area (Table S1). A total of 43 of the 50 proteins with the highest areas were OM proteins, predicted lipoproteins, flagellar proteins, periplasmic proteins, or predicted secreted proteins. Taken together, these data indicated that the flagellar preparation was highly enriched for proteins from the flagellum, sheath, OM, and periplasmic space. HP0135 (designated as hypothetical protein CV725_02940 in *H. pylori* B128) is a small predicted lipoprotein that had one of the highest peptide mass spectrum peak areas. The HP0135 prolipoprotein is only 44 amino acid residues in length, and following cleavage of the signal peptide, the mature HP0135 lipoprotein is only 28 amino acid residues in length.

The presence of HP0135 in the flagellar sheath preparation suggested the protein is sorted to the OM by the Lol pathway. Consistent with this observation, HP0135 was reported to be enriched in OMVs generated by *H. pylori* 26695 [35]. To investigate the localization of HP0135 further, we isolated OM proteins from *H. pylori* B128 using a differential detergent extraction procedure and identified the resulting proteins by mass spectrometry. *H. pylori* membranes were resuspended in a buffer containing Sarkosyl to solubilize the inner membrane and hybrid membrane vesicles, followed by high-speed centrifugation to pellet the OM (Sarkosyl-insoluble) fraction. A total of 108 proteins with a high protein FDR confidence were identified by mass spectrometry in the Sarkosyl-insoluble fraction (Table S2). A total of 39 of the proteins identified in the Sarkosyl-insoluble fraction (36% of the total proteins) were known or predicted OM proteins, whereas only 12 inner membrane proteins (11% of the total proteins) were identified in the sample (Table S2), indicating the Sarkosyl-insoluble fraction was enriched for OM proteins. Many of the proteins identified in the Sarkosyl-insoluble fraction were known periplasmic proteins or were predicted periplasmic proteins based on possessing a predicted Sec/SPI signal peptide (Table S2). The periplasmic proteins were likely trapped inside OMVs that were generated following lysis of the *H. pylori* cells with the French press. Thirteen known or predicted lipoproteins were identified in the Sarkosyl-insoluble fraction (Table S2), one of which was HP0135, which provides supporting evidence for the localization of HP0135 to the OM.

**Figure 1:**
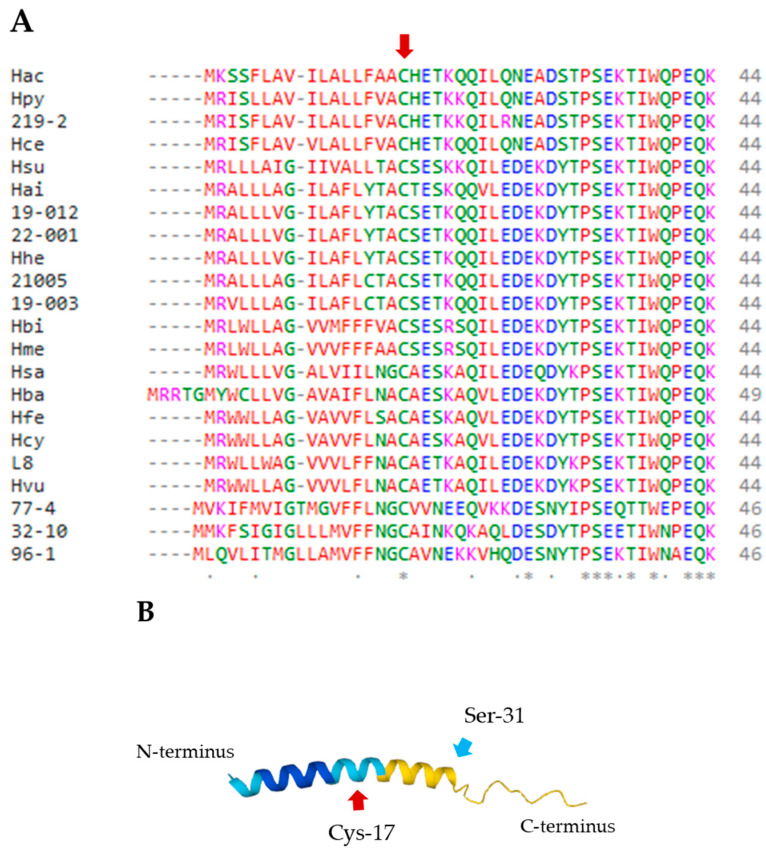
Alignment of the amino acid sequences of HP0135 homologs and the predicted tertiary structure of HP0135. (**A**) Amino acid sequences of HP0135 homologs were aligned using the Clustal Omega (www.ebi.ac.uk/jdispatcher/msa/clustalo (accessed on 10 September 2024)) sequence analysis tool [38]. HP0135 homologs are from the following species: *Helicobacter acinonychis* (Hac), *H. pylori* (Hpy), *Helicobacter* sp. 219-2 (219-2), *Helicobacter cetorum* (Hce), *Helicobacter suis* (Hsu), *Helicobacter ailurogastricus* (Hai), *Helicobacter* sp. NHP19-012 (19-012), *Helicobacter* sp. NHP22-001 (22-001), *Helicobacter heilmannii* ASB1.4 (Hhe), *Helicobacter* sp. NHP21005 (21005), *Helicobacter* sp. NHP19-003 (19-003), *Helicobacter bizzozeronii* (Hbi), *Helicobacter mehlei* (Hme), *Helicobacter salmonis* (Hsa), *Helicobacter baculiformis* (Hba), *Helicobacter felis* (Hfe), *Helicobacter cynogastricus* (Hcy), *Helicobacter* sp. L8 (L8), *Helicobacter vulpis* (Hvu), *Helicobacter* sp. 13S00477-4 (77-4), *Helicobacter* sp. 12S02232-10 (32-10), and *Helicobacter* sp. 11S02596-1 (96-1). For each amino acid position, identical amino acids are indicated with an asterisk and similar amino acids are indicated with one dot. Hydrophobic, polar, basic, and acidic amino acid residues are indicated in red, green, magenta, and blue, respectively. The predicted signal peptide cleavage site is indicated by the red arrow. (**B**) Tertiary structure of HP0135 prolipoprotein predicted by AlphaFold2 [39]. Regions in blue indicate a high confidence for predicted structure, while regions in yellow indicate a lower confidence for predicted structure. The position of Cys-17, which is the site of cleavage, is indicated. The position of Ser-31, which is the last amino acid residue within a well-ordered tertiary structure, is also indicated.

A PSI-BLAST (Position-Specific Iterated BLAST) analysis of the NCBI non-redundant protein database (accessed on 13 November 2024) and a blastp analysis of the JGI Integrated Microbial Genomes and Microbiomes (IMG/M) database (accessed on 6 February 2024) identified HP0135 homologs in 21 additional gastric *Helicobacter* species that colonize various domesticated and wild mammals [36,37]. A tblastn analysis of the NCBI core nucleotide database (accessed on 13 November 2024) and a blastn analysis of the JGI IMG/M database (accessed on 13 November 2024) failed to identify any additional HP0135 homologs within the genus *Helicobacter*. HP0135 homologs were not identified in any bacterial species outside the genus *Helicobacter*. Alignment of the HP0135 homologs revealed that the C-terminal regions of the proteins are highly conserved, with 8 of the last 12 amino acid residues being identical and two more residues being similar in all 22 protein sequences (Figure 1A).

A tertiary structure of HP0135 predicted by AlphaFold2 [39] indicated that the 13 amino acid residues at the C-terminus of the protein are disordered (Figure 1B). Intrinsically disordered polypeptide segments do not adopt well-folded three-dimensional structures but instead assume a variety of interconverting conformations [40]. These intrinsically disordered polypeptide segments lack sufficient hydrophobic amino acid residues to mediate cooperative folding and usually contain a high proportion of polar and charged amino acids [41]. Indeed, 9 of the 13 amino acids in the predicted disordered region of HP0135 are polar or charged, and only 4 of the amino acid residues are hydrophobic.

### 2.2. The H. pylori Δhp0135 Mutant Produces High Amounts of OMVs

**Figure 2:**
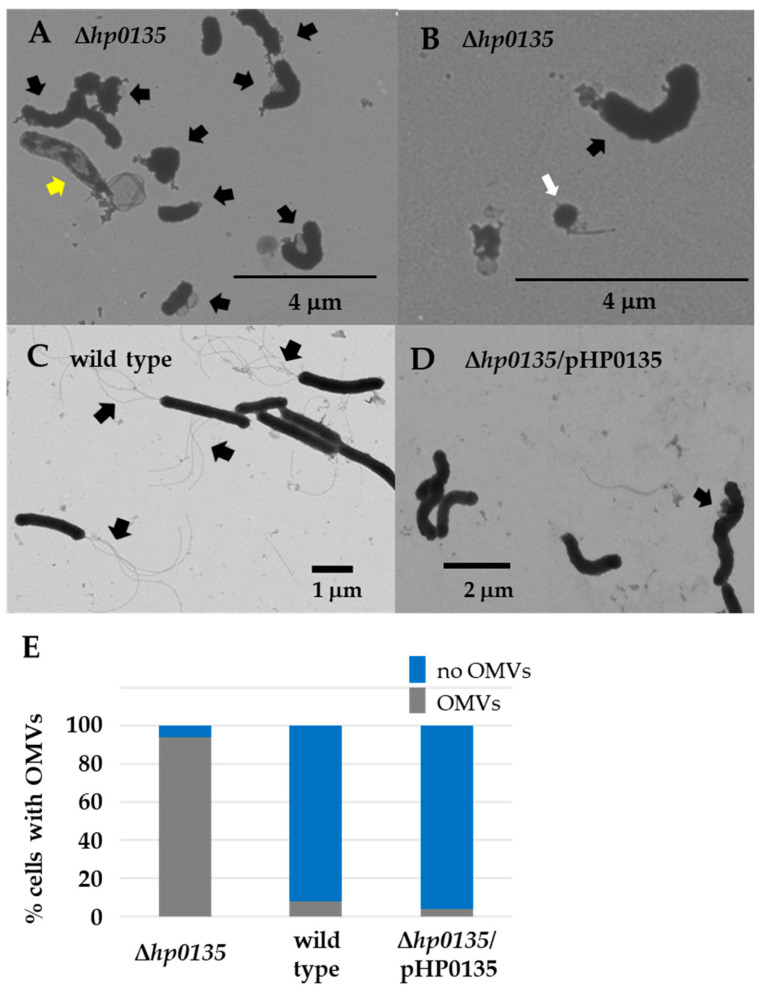
TEM images of wild-type *H. pylori* B128, the original Δ*hp0135* mutant, and the complemented Δ*hp0135* strain. (**A**) TEM field showing examples of *H. pylori* Δ*hp0135* mutant cells. The black arrows indicate cells in the field that have OMVs. The yellow arrow indicates a cell that appears to be undergoing lysis. (**B**) An example of a mini-cell formed by the *H. pylori* Δ*hp0135* mutant is indicated by the white arrow. The black arrow indicates a normal-length cell that displays OMVs at the cell pole. (**C**) TEM field showing wild-type *H. pylori* B128 cells. Polar flagella are indicated by the black arrows. (**D**) TEM field showing examples of cells of Δ*hp0135* mutant that was complemented with a plasmid-borne copy of *hp0135*. The arrow indicates a cell that has OMVs. (**E**) Proportion of cells with OMVs in Δ*hp0135* mutant, wild-type *H. pylori* B128, and Δ*hp0135*/pHP0135 strain. Cells were counted from two biological replicates for each strain, and the number of cells counted for the Δ*hp0135* mutant, wild type, and Δ*hp0135*/pHP0135 strain were 139, 78, and 60, respectively. The proportion of cells with OMVs in the Δ*hp0135* mutant was significantly higher compared to the wild type and the complemented strain as determined by Fisher’s exact test (*p*-value < 0.00001).

To examine the potential physiological role of HP0135, we deleted *hp0135* in *H. pylori* B128 and characterized the resulting mutant. Since HP0135 appeared to be a prevalent component of the flagellar sheath, cells of the Δ*hp0135* mutant were grown to the mid-log phase and examined by transmission electron microscopy (TEM) to determine if the absence of HP0135 impacted the morphology of the flagellar sheath. Unexpectedly, the cells of the Δ*hp0135* mutant were aflagellated (Figure 2A,B). In contrast, most of the wild-type cells were flagellated (Figure 2C). In addition to lacking flagella, cells of the Δ*hp0135* mutant produced large amounts of OMVs compared to wild type (Figure 2). Mini-cells were also frequently observed in the TEM fields of the Δ*hp0135* mutant (Figure 2B). Mini-cells result from cell division occurring near the cell pole cell instead of the normal position at the mid-cell and are non-viable since they do not receive a copy of the chromosome. Approximately 4% of the Δ*hp0135* cells were mini-cells, which we defined as spherical cells that were <0.5 μm in diameter. In contrast, mini-cells were rarely observed for wild type or cells of the complemented strain. Introducing a plasmid carrying *hp0135* (plasmid pHP0135) into the Δ*hp0135* mutant suppressed the hypervesiculation phenotype of the Δ*hp0135* mutant, as only ~3% of the cells of the complemented strain produced OMVs (Figure 2E). Taken together, these findings strongly suggest the loss of HP0135 was responsible for the high amount of OMVs produced by the Δ*hp0135* mutant as well as the increase in mini-cell formation in the mutant.

### 2.3. Examination of the Motility Defect of the Δhp0135 Mutant

While the plasmid-borne copy of *hp0135* suppressed the hypervesiculation phenotype of the Δ*hp0135* mutant, it failed to rescue the flagellation defect of the strain (Figure 2D). This finding suggested the Δ*hp0135* mutant had a secondary mutation that was responsible for the flagellation defect. Whole genome sequencing of the Δ*hp0135* mutant revealed a frameshift mutation in *fliP* that resulted from the loss of a nucleotide within a homopolymeric tract of eight cytidines (Table 1). FliP is a component of the flagellar type III secretion system that transports axial components of the flagellum, such as the rod, hook, and filament proteins, across the inner membrane [42]. Josenhans and co-workers had previously reported that changes in the length of this poly(C)-tract in *H. pylori* 26695 that presumably resulted from slipped-strand mispairing-mediated mutagenesis suggested that altering the length of the poly(C)-tract in *fliP* was a mechanism that *H. pylori* used to switch between “on” and “off” phases for flagellation and motility [43]. The frameshift mutation in *fliP* presumably accounted for the aflagellation of the Δ*hp0135* mutant.

In addition to the frameshift mutation in *fliP*, the Δ*hp0135* mutant had a missense mutation in *mltD*, which encodes a putative lytic transglycosylase (Table 1). Lytic transglycosylases are periplasmic peptidoglycan hydrolases that cleave the glycan backbone of peptidoglycan to generate anhydromuropeptides [44]. The missense mutation in *mltD* altered the tyrosine at position 207 to a histidine residue. The Δ*hp0135* mutant also had a single guanosine deletion within a poly(G)-tract in the pseudogene *pldA*. PldA is an integral OM phospholipase that degrades glycerophospholipids and lysoglycerophospholipids by removing an acyl chain from the *sn*-1 of the *sn*-2 position [45,46]. The substrate-binding pocket of PldA is at the cell surface, which allows the enzyme to degrade selectively glycerophospholipids in the outer leaflet and thereby assist in maintaining the lipid asymmetry of the OM [47,48]. *H. pylori pldA* undergoes phase variation as a result of reversible changes in the length of the poly(G)-tract where the insertion occurred in the Δ*hp0135* mutant, which manifests in the translation of full-length, active PldA or a truncated, inactive enzyme [49]. In the wild-type *H. pylori* B128 strain used in our study, the poly(G)-tract in *pldA* is ten nucleotides in length, which results in *pldA* being in the “off” phase. In the Δ*hp0135* mutant, the length of the poly(G)-tract in *pldA* was reduced to nine nucleotides, but the gene remained in the “off” phase (Table 1).

**Figure 3:**
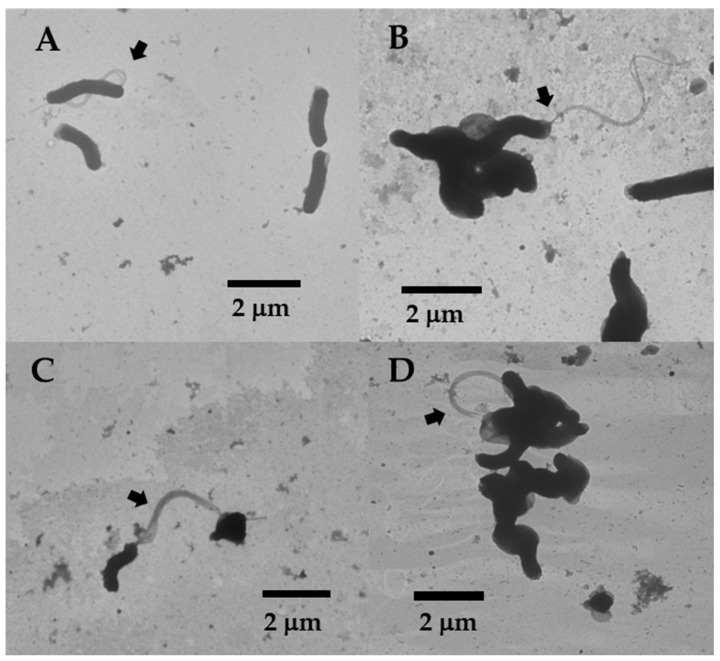
TEM images of strains RIH3A and RIH3C. (**A**,**B**) TEM images of strain RIH3A. The black arrows indicate flagellated cells. (**C**,**D**) TEM images of strain RIH3C. The black arrows indicate flagellated cells.

Inoculating the Δ*hp0135* mutant into a soft agar medium occasionally produced a small swim halo after a few days. Swim halos are formed by cells migrating from the point of inoculation in the soft agar medium and multiplying in the newly colonized area. Two independently isolated motile variants of Δ*hp0135* mutant were isolated by picking cells from the edge of a small swim halo and inoculating the cells into fresh soft agar. The inoculated cells formed a swim halo that was larger than the original swim halo, and cells were picked from the edge of the swim halo and inoculated into fresh, soft agar. After repeating the process a third time, clonal isolates were obtained by streaking the cells from the edge of the swim halo on the TSA-HS medium. The motility in soft agar was confirmed for two isolates, which we designated as strains RIH3A and RIH3C. Examination of strains RIH3A and RIH3C by TEM revealed that only about 20% of the cells were flagellated, and these cells possessed one or two flagella (Figure 3). This contrasted with the wild-type strain, where the vast majority of cells were flagellated, and most of the cells had three or four flagella. Whole genome sequencing of strains RIH3A and RIH3C did not reveal mutations in *fliP* above the 80% frequency used as the threshold for calling mutations, which suggested the poly(C)-tract in *fliP* had reverted to eight cytidines (i.e., reverted to the wild-type *fliP* allele) in these strains. Examining the marginal predictions for mutations in the two strains (i.e., mutations that were below the 80% frequency cutoff), however, revealed that a significant proportion of cells in the two strains had predicted mutations in *fliP* that would have interfered with the expression of full-length FliP. In RIH3C, only ~36% of the reads for the region that included the poly(C)-tract in *fliP* (a total of 231 reads) had eight cytidines, which would have resulted in *fliP* switching to the ‘on’ phase. The poly(C)-tract in *fliP* for most of the other reads for this region (~57%) had nine cytidines, which resulted in *fliP* remaining in the ‘off’ phase. In RIH3A, ~36% of the reads (a total of 286 reads) were predicted to have a 43-bp duplication at nucleotide position 321 in *fliP*, which would have resulted in a frameshift mutation. These *fliP* mutations in RIH3A and RIH3C presumably arose during their propagation and likely contributed to the high proportion of aflagellated cells that were observed for these strains (Figure 3).

Strains RIH3A and RIH3C had two mutations that were not present in the original Δ*hp0135* mutant (Table 1). One of the mutations was a deletion of a single cytosine within a poly(C)-tract upstream of *CV725_RS08295*, which is predicted to encode a 72-amino-acid hypothetical protein. The corresponding ORF in the annotated *H. pylori* B128 genome in the JGI IMG/M database is predicted to encode a 116-amino-acid hypothetical protein and contains the poly(C)-tract. In *H. pylori* 26695, the poly(C)-tract is within the *hp1353*, which is upstream of the *CV725_RS08295* homolog. Thus, the organization of the genes in this region of the *H. pylori* B128 genome is unclear. Nevertheless, the single nucleotide deletion occurred in RIH3A, and RIH3C is predicted to allow expression of the 116-amino-acid protein predicted from the annotation of the *H. pylori* B128 genome in the JGI IMG/M database. RIH3A and RIH3C also had mutations in *fur*, which encodes the ferric uptake regulator. *H. pylori* Fur regulates the expression of genes involved in iron homeostasis, but it also controls the expression of genes involved in a variety of processes and, therefore, functions as a master regulator of gene expression [50]. In RIH3A, the mutation in *fur* was an insertion of a single adenosine within a poly(A)-tract that results in a frameshift mutation early in the coding sequence (Table 1). Strain RIH3C had a missense mutation in *fur* that resulted in a replacement of valine at position 140 with a methionine residue (Table 1). Strains RIH3A and RIH3C also had a two-nucleotide deletion within the poly(G)-tract in *pldA* that reduced the length of the poly(G)-tract to eight nucleotides and resulted in the gene switching to the “on” phase (Table 1).

### 2.4. HP0135 Is Not Linked to the Murein Sacculus

*E. coli* Lpp, also known as Braun’s lipoprotein or murein lipoprotein, stabilizes the OM by anchoring it to the peptidoglycan layer [51]. Lpp is covalently attached to peptidoglycan via a peptide bond formed between the ε-amino group of lysine at the C-terminus of Lpp and a carboxyl group of *meso*-diaminopimelate in the peptidoglycan tetrapeptide by one of three L,D-transpeptidases [52]. HP0135 and Lpp have some similarities. For example, similar to the Δ*hp0135* mutant, cells of the *E. coli* Δ*lpp* mutant form large amounts of OMVs [53]. Secondly, HP0135 and Lpp are both small lipoproteins, with the mature lipoproteins consisting of 28 and 58 amino acids, respectively. Thirdly, HP0135 homologs have a conserved, C-terminal lysine (Figure 1A) that has the potential to be crosslinked to peptidoglycan as occurs with Lpp. Given the parallels between Lpp and HP0135, we considered that similar to Lpp, HP0135 may stabilize the OM by anchoring it to the peptidoglycan layer. To examine this hypothesis, we isolated the murein sacculus from wild-type *H. pylori* B128 and identified the proteins associated with the sacculus by mass spectrometry following trypsin digestion. Eighteen proteins with a high protein FDR confidence were identified from the murein sacculus preparation, but HP0135 was not one of these proteins (Table S4). In the isolated flagella sample, three peptides derived from HP0135 following trypsin digestion (Q^23^ILQNEADSTPSEK^36^, K^22^QILQNEADSTPSEK^36^, and T^37^IWQPEQK^44^) were identified by mass spectrometry, and if HP0135 had been covalently linked to peptidoglycan, we should have observed each of these peptides in the murein sacculus preparation. Thus, despite the parallels between HP0135 and Lpp, the two proteins do not appear to be functionally equivalent. The failure to detect HP0135 in the murein sacculus preparation was not entirely unexpected since *H. pylori* lacks homologs of the L,D-transpeptidases (ErfK, YcfS, and YbiS) that link Lpp to murein in *E. coli* [54].

## 3. Discussion

We report here that loss of the small lipoprotein HP0135 in *H. pylori* B128 results in the cells producing large amounts of OMVs during logarithmic growth (Figure 2). OMVs are formed as a part of the normal growth of bacteria and have a wide range of physiological roles, including intracellular and extracellular communication, toxin delivery, modulating host immune response, antibiotic resistance, interbacterial killing, quorum sensing, stress responses, horizontal gene transfer, and polysaccharide digestion (reviewed in [31,55,56]. The production of OMVs in *H. pylori* is dependent on the growth phase [35,57]. Cells of *H. pylori* strain CCUG17875 formed few OMVs during logarithmic growth, but OMV production increased as cultures entered the stationary phase, and cells in the late stationary phase produced large amounts of OMVs [57]. The same trend in OMV production was observed in *H. pylori* 26695 as cultures progressed from the early logarithmic phase to the stationary phase [35]. Thus, while *H. pylori* normally produces OMVs, the elevated amounts of OMVs generated by the Δ*hp0135* mutant during logarithmic growth are abnormal. In addition to the hypervesiculation phenotype, the Δ*hp0135* mutant displayed an increased tendency to produce mini-cells. Loss of HP0135 appeared to be responsible for the hypervesiculation and increased frequency of mini-cells in the Δ*hp0135* mutant, as introducing a plasmid-borne copy of *hp0135* suppressed the aberrant phenotypes of the strain (Figure 2). Thus, HP0135 may be added to the growing list of membrane-associated small proteins that have important roles in cellular functions.

Small proteins in bacteria that are associated with the membrane often produce their effects on cellular functions by binding to larger proteins. The binding of small membrane proteins to their binding partners can impact the larger proteins in a variety of ways, including their recruitment to the membrane, alteration in their stability, their assembly into protein complexes, or modulation of their activity [28,29,30]. Alternatively, a number of membrane-associated small proteins are members of toxin-antitoxin systems, some of which disrupt membranes by forming pores [29].

Small membrane proteins that have been studied to date have been primarily, if not exclusively, associated with the inner membrane. In contrast, HP0135 is predicted to localize to the OM based on the previous report that it is enriched in OMVs [35], as well as our findings that HP0135 was prevalent in the proteome of the flagella and flagellar sheath sample (Table S1) and was present in the Sarkosyl-insoluble fraction of *H. pylori* membranes (Table S2). Despite its localization to the OM, it is likely that HP0135 mediates its effects on OMV production and mini-cell formation through one of the general mechanisms used by small proteins associated with the inner membrane to influence cellular processes. Specifically, we hypothesize that HP0135 binds to one or more larger proteins, and these protein-protein interactions affect the localization, stability, or activity of the larger protein. Thus, identifying HP0135 binding partners will be crucial for understanding how the loss of HP0135 results in increased OMV production and mini-cell formation.

In addition to identifying HP0135 binding partners, understanding the mechanisms involved in OMV biogenesis is essential for determining how the loss of HP0135 results in hypervesiculation. While there is no universal mechanism to account for OMV formation, various models for OMV biogenesis have been proposed [31,58]. A number of studies have shown the loss of proteins that anchor the OM to the peptidoglycan allows the membrane to bend and form vesicles. In *E. coli* and other bacteria, covalent crosslinking between Lpp and peptidoglycan stabilizes the OM, and loss of Lpp results in hypervesiculation [53]. The OM proteins OmpA and Pal also help to stabilize the OM through non-covalent interactions with the underlying peptidoglycan layer, and the loss of these proteins results in increased OMV production in various bacterial species [59,60,61,62]. Another model for OMV biogenesis proposes that the accumulation of peptidoglycan fragments or misfolded proteins in the periplasm exerts turgor pressure on the OM to induce vesicle formation [31,58]. Changes in the content and distribution of lipids within the OM that increase the curvature of the membrane also induce vesicle formation [31,58]. For example, the localized enrichment of LPS species with negatively charged O-antigen chains in *Pseudomonas aeruginosa* is thought to increase the curvature of the membrane and promote vesicle formation [63]. Similarly, disrupting the ATP-binding cassette (ABC) transport system that is involved in the retrograde transport of phospholipids from the OM results in the accumulation of phospholipids in the outer leaflet of the OM, which increases the curvature of the membrane and leads to vesicle formation [64,65].

Since we were unable to detect HP0135 in the murein sacculus preparation (Table S3) and HP0135 does not possess a known peptidoglycan-binding domain (e.g., LysM domain, PGBD superfamily, SPOR domain), it seems unlikely that HP0135 stabilizes the OM by anchoring it to the underlying peptidoglycan layer. The other models of OMV biogenesis, however, seem plausible in accounting for the hypervesiculation phenotype of the Δ*hp0135* mutant. For example, *H. pylori* has multiple peptidoglycan hydrolases that are involved in modifying the murein sacculus [66,67,68,69], and HP0135 may regulate the activity of one or more of these enzymes. If that is the case, the unregulated activity of the peptidoglycan hydrolase in the absence of HP0135 might result in peptidoglycan fragments accumulating in the periplasmic space and inducing vesicle formation. Alternatively, HP0135 may regulate the activity of a protein involved in the maintenance of phospholipid homeostasis in the OM, and loss of HP0135 may disrupt this homeostasis, which would lead to vesicle formation.

The OM is an asymmetrical lipid bilayer in which the inner leaflet is composed of glycerophospholipids, and the outer leaflet is populated primarily by LPS. The OM is an effective barrier to bile salts, antibiotics, and other noxious compounds, and the lipid asymmetry of the OM is critical for its barrier function. The OM phospholipase assists in maintaining the lipid asymmetry of the OM by hydrolyzing glycerophospholipids in the outer leaflet of the OM into lysophospholipids and fatty acids [47,48,70]. *H. pylori pldA* undergoes phase variation as a result of slippage during DNA replication in a poly-(G) tract [49]. Surveying nearly 800 *H. pylori* genomes in the JGI IMG/M database (accessed on 6 November 2024), we found that *pldA* is in the “off” phase in ~12% of strains, including *H. pylori* B128, which was the strain used in this study. In the original Δ*hp0135* mutant, the length of the phase-variable poly(G)-tract in *pldA* was reduced from ten to nine nucleotides, but *pldA* remained in the “off” phase (Table 1). In the two motile variants of the Δ*hp0135* mutant, though, *pldA* was switched from the “off” to the “on” phase (Table 1). There may have been selective pressure for switching *pldA* to the “on” phase in the motile isolates of the Δ*hp0135* mutant due to the disruption in OM homeostasis that likely resulted from the elevated production and subsequent shedding of OMVs. Shedding of OMVs by the motile variants of the Δ*hp0135* mutant may have been promoted by flagellar rotation, which may have accounted for why *pldA* remained in the “off” phase in the original, aflagellated Δ*hp0135* mutant. If flagellar rotation did facilitate the shedding of OMVs, this may have exerted selective pressure for the switching of *fliP* to the “off” phase in the original Δ*hp0135* mutant.

In addition to the phase variation switch in *pldA*, the motile variants of the Δ*hp0135* mutant had mutations in *CV725_RS08295* and *fur* that were not present in the Δ*hp0135* mutant (Table 1). Since it is unclear if *CV725_RS08295* encodes a functional protein, it seems likely that the mutation in this gene had little impact on the motile variants of the Δ*hp0135* mutant. On the other hand, *H. pylori* Fur regulates genes involved in a variety of processes and responds not only to intracellular iron levels but also to oxidative stress and changes in pH [50]. *H. pylori* Fur is also atypical compared to Fur proteins from many other bacteria in that it represses and activates genes in both the iron-bound (holo-Fur) and apo (apo-Fur) forms of the protein [50]. *H. pylori* Fur contains three distinct metal-binding sites, which are designated as S1, S2, and S3. In RIH3A, the mutation in *fur* was an insertion of a single adenosine within a poly(A)-tract that results in a frameshift mutation early in the coding sequence (Table 1). Strain RIH3C had a missense mutation in *fur* that resulted in the substitution of methionine at Val-140 (Table 1). Val-140 is located at the end of β-strand 5 (β5) of the protein, which also contains His-134, one of the amino acid residues that coordinate the metal ion in S3 [71]. Gilbreath and co-workers identified three *H. pylori fur* mutations in β5 of the protein (I132N, D135Y, and M138T) that phenocopied the Δ*fur* mutant for *amiE* regulation, which suggested these amino acid residues are involved in proper metal ion coordination [72]. The expression of a number of flagellar genes, including *flaB* (encodes a minor flagellin), *flgK* (encodes a hook-associated protein), and *fliY* (encodes a C-protein), was reported to be downregulated in an *H. pylori* G27 Δ*fur* mutant [73]. Thus, the mutations in *fur* in RIH3A and RIH3C may have contributed to the reduced flagellation of these strains (Figure 3). Moreover, if flagellar rotation promoted the shedding of OMVs in the motile variants of the Δ*hp0135* mutant, this may have exerted selective pressure for mutations in *fur* that reduced flagellation in these strains.

## 4. Materials and Methods

### 4.1. Bacterial Strains and Growth Conditions

Procedures for cloning and plasmid construction were conducted using *E. coli* Turbo (New England Biolabs, Ipswitch, MA, USA). Lysogeny broth (LB) and LB agar medium were used to culture *E. coli* strains. The LB media were supplemented with kanamycin (30 μg/mL), ampicillin (100 μg/mL), isopropyl β-D-1-thiogalactopyranoside (IPTG; 0.1 mM), or 5-bromo-4-chloro-3-indolyl β-D-galactopyranoside (x-gal; 40 μg/mL) as required. *H. pylori* B128, which was kindly provided by Richard M. Peek, Jr., was the parental strain for the *H. pylori* strains described in this study. For the growth of *H. pylori* cultures on a solid medium, cells were grown at 37 °C on tryptic soy agar supplemented with 5% heat-inactivated horse serum (TSA-HS) under an atmosphere consisting of 10% CO_2_, 6% O_2,_ and 84% N_2_. For culturing *H. pylori* in a liquid medium, cells were grown in brain heart infusion (BHI) medium supplemented with 5% heat-inactivated horse serum with shaking in sealed bottles under an atmosphere of 5% CO_2_, 10% H_2_, 10% O_2_, and 75% N_2_. Kanamycin (30 μg/mL) and sucrose (5% wt/vol) were added to the growth medium for *H. pylori* cultures as needed. To assess the motility of *H. pylori* strains, cells were inoculated into a soft agar medium consisting of Mueller–Hinton broth, 10% heat-inactivated horse serum, 20 mM 2-(N-morpholino) ethanesulfonic acid (MES; pH 6.0), and 0.4% noble agar [74]. Chemicals and reagents used for culturing bacteria were purchased from Thermo Fisher Scientific, Waltham, MA, USA.

### 4.2. Constructing the H. pylori Δhp0135 Mutant

Genomic DNA (gDNA) from *H. pylori* B128 genomic DNA (gDNA) served as the template for PCR and was purified using the Wizard genomic DNA purification kit (Promega, Madison, WI, USA). A 355-bp region upstream and a 505-bp region downstream of the *hp0135* homolog were amplified from *H. pylori* B128 gDNA using PrimeSTAR DNA polymerase (Takara Bio, San Jose, CA, USA) together with the primer pairs HP0135_US_F (5′-TTTTTTAATCGCTGACATGCTTAAA-3′)/HP0135_US_R (5′-GAATTCGATTATCCTCGAGATTACAGCTAAAAGAGAAATTCTCA-3′) and HP0135_DS_F (5′-GATAATCGAATTCGCTAGCAACCATCTGGCAACCTGAACAAAAA-3′)/HP0135_DS_R (5′- CCTGATAACGCTCAATCGCATCAAA-3′), respectively. Primers HP0135_US_R and HP0135_DS_F were complementary and contained sequences for Xho1 and Nhe1 restriction sites. All primers used in the study were purchased from Integrated DNA Technologies, Coralville, IA, USA. Overlapping PCR using Phusion DNA polymerase (New England Biolabs, Ipswich, MA, USA) was used to join the resulting amplicons, and the resulting amplicon was incubated with Taq DNA polymerase (Promega, Madison, WI, USA) to add A-overhangs to the 3′ ends of the amplicon, which was then ligated into pGEM-T Easy (Promega) to generate plasmid pRI2. The sequence of the inserted region in pRI2 was confirmed by DNA sequencing (Eton Biosciences, Research Triangle Park, NC, USA). A kan^R^-*sacB* cassette from plasmid pJC038 [74] was cloned into the Xho1 and Nhe1 sites of plasmid pRI2 to generate plasmid pRI3.

Plasmid pRI3, which served as a suicide vector for knocking out the *hp0135* homolog, was introduced into *H. pylori* B128 by natural transformation as described [74]. Kanamycin-resistant isolates were checked by PCR using the primer pair HP0135_US_F/HP0135_DS_R to verify that the kan^R^-*sacB* cassette had integrated into the *hp0135* locus. One of the isolates in which *hp0135* was replaced with the kan^R^-*sacB* cassette was designated strain RI1 and was transformed with the suicide vector pRI2 to replace the kan^R^-*sacB* cassette with an unmarked deletion of *hp0135.* Transformants in which the kan^R^-*sacB* cassette was replaced with the unmarked deletion were counter-selected on TSA-HS supplemented with 5% sucrose as described [75]. Sucrose-resistant isolates were screened for kanamycin sensitivity on TSA-HS supplemented with kanamycin, and deletion of *hp0135* in the isolates was confirmed by PCR using primer pair HP0135_US_F/HP0135_DS_R and DNA sequencing of the resulting amplicon. One of the kanamycin-sensitive isolates in which it was verified that *hp0135* was deleted was designated as strain RI2 and stored at −70 °C.

### 4.3. Complementation of the H. pylori Δhp0135 Mutation

A DNA sequence that included the coding region of *hp0135* along with 311 bp and 38 bp of upstream and downstream sequence, respectively, was amplified from *H. pylori* B128 gDNA using PrimeSTAR DNA polymerase and the primers HP0135_fwd (5′GCTAGCTTAAAAAACAACGATCTTGATTGAA-3′) and HP0135_rev (5′GGATCCGTTATTTTAAAAAATGCCTTTGAGG-3′). Primers HP0135_fwd and HP0135_rev introduced NheI and BamHI sites, respectively, which were used for a subsequent cloning step. A-overhangs were added to the 3′ ends of the amplicon using Taq DNA polymerase, after which the amplicon was ligated into pGEM-T Easy. Insertion of the expected DNA sequence into the plasmid was confirmed by restriction enzyme digestion and DNA sequencing, and the resulting plasmid was designated pRI1. Plasmid pRI1 was digested with NheI and BamHI, and the DNA fragment bearing the *hp0135* sequence was cloned into the shuttle vector pHel3 [76] to create plasmid pRI4, which was transformed into the *H. pylori* B128 Δ*hp0135* mutant (strain RPI3) to create strain RI4.

### 4.4. Transmission Electron Microscopy

Liquid cultures of *H. pylori* were grown for 12–16 h to mid-log phase. Cells from the cultures were collected by centrifugation, fixed with formaldehyde and glutaraldehyde, and negatively stained with uranyl acetate as described [74]. At least three biological replicates were conducted for each *H. pylori* strain. Cells were visualized using a JEOL JEM 1011 transmission electron microscope (Tokyo, Japan) operated at 80 kV. Cell length measurements were conducted using Image J version 1.54i.

### 4.5. DNA Sequencing and Analysis

gDNA from the *H. pylori* strains was purified using the Wizard genomic DNA purification kit (Promega, Madison, WI, USA) and submitted to the SeqCenter (Pittsburgh, PA, USA) for genomic library preparation and Illumina sequencing. DNA reads were mapped using the *breseq* computational pipeline [77] with the *H. pylori* B128 genome sequence in the NCBI database (Accession no.: NZ_CP024951.1).

### 4.6. Isolation of H. pylori Flagella

Cells of *H. pylori* B128 grown on TSA-HS medium were harvested and resuspended in phosphate-buffered saline (PBS). Flagella were sheared from the cells by vortexing the cell suspension for 60 s. Cells were pelleted by centrifugation at 7310× *g* for 10 min, and the resulting supernatant was passed through a 0.4 µm filter to remove any cells that had not been pelleted. The resulting filtrate was subjected to centrifugation at 106,000× *g* for 30 min to pellet the flagella, which were then resuspended in PBS.

### 4.7. Enrichment of H. pylori Outer Membrane Proteins

*H. pylori* OM proteins were enriched by adopting a differential detergent extraction procedure that was originally described for the isolation of *C. jejuni* OM proteins [78]. *H. pylori* cells grown on TSA-HS medium were harvested and resuspended in 4 mL of 10 mM sodium phosphate buffer, pH 7.0, containing 5 mM MgSO_4_. Cells were lysed using a Cell Disruptor System (Constant Systems Ltd., Daventry, UK) at 20,000 psi. The cell lysate was subjected to centrifugation at 5600× *g* for 20 min to pellet the unbroken cells and cell debris. The resulting supernatant was centrifuged at 311,000× *g* for 40 min to pellet the membranes. The membrane pellet was resuspended in 1% Sarkosyl and incubated for 20 min at room temperature to solubilize the inner membrane and hybrid membrane vesicles. The membrane suspension was centrifuged at 311,000× *g* for 40 min, and the resulting supernatant was saved as the Sarkosyl-soluble fraction. The pellet was washed by resuspending it in 1% Sarkosyl, followed by centrifugation at 311,000× *g* for 40 min. The supernatant was removed and discarded, and the pellet was washed a second time by resuspending it in 1% Sarkosyl and then centrifuging at 311,000× *g* for 40 min. The resulting pellet was resuspended in 200 µL in 1% Sarkosyl. Chemicals used for enrichment of OM proteins were purchased from Thermo Fisher Scientific, Waltham, MA, USA.

### 4.8. Isolation of Murein Sacculus

*H. pylori* B128 cells grown on TSA-HS medium were harvested and resuspended in 4 mL of PBS buffer. The cells were lysed using a Cell Disruptor System at 20,000 psi. The lysed cells were transferred into a glass tube with a magnetic stir bar. The tube was placed in a boiling water bath, and 1.5 mL of 5% sodium dodecyl sulfate (SDS) solution was added slowly to the mixture as it was stirred. The sample was boiled for 30 min to two hours, then incubated overnight at room temperature with stirring. The sample was centrifuged at 150,000× *g* for 10 min at room temperature. The resulting pellet was washed three times with water, followed by centrifugation at 150,000× *g* for 10 min to remove the SDS, then resuspended in 900 µL of water.

### 4.9. Proteomic Analysis of H. pylori Samples

Proteins of *H. pylori* flagella preparations and Sarkosyl-insoluble fraction from *H. pylori* membranes (2 to 5 μg total protein) were loaded onto an SDS-polyacrylamide gel and subjected to electrophoresis until the proteins started to enter the resolving gel. Gel slices containing the proteins were removed and sent to the University of Georgia Proteomics and Mass Spectrometry (UGA PAMS) facility for protein identification. The protocol for the in-gel trypsin digestion and peptide extraction is available on the UGA PAMS website (https://pams.uga.edu/protocols/ (accessed on 26 December 2024)). Proteins in the gel slices were digested with trypsin, and the resulting peptides were analyzed by LC-MS/MS with a ThermoScientific Orbitrap Velo Elite mass spectrometer (Thermo Fisher Scientific, Waltham, MA, USA) coupled with nano-HPLC using a 90 min elution gradient. Mass spectrometry data were searched against a protein database using the Mascot Server 2.8.0 search engine (Matrix Science, London, UK) for protein identification. For identification of proteins associated with the murein sacculus, the isolated sacculus (5 μg) was digested with trypsin, and the resulting peptides were analyzed as described above for the flagella and OM protein preparations.

## 5. Conclusions

*H. pylori* HP0135 is a small lipoprotein that is predicted to localize to the OM. We show here that deletion of *hp0135* in *H. pylori* B128 results in hypervesiculation and increased mini-cell formation. However, it is not known how the loss of HP0135 results in hypervesiculation and increased mini-cell formation; small membrane proteins in bacteria generally elicit their effects on cellular functions through interactions with larger proteins. Therefore, identifying proteins that interact with HP0135 will provide valuable clues on how this small lipoprotein impacts OMV formation and cell division in *H. pylori*.

## Figures and Tables

**Table 1:** Identified single nucleotide polymorphisms and indels in *H. pylori* B128 Δ*hp0135* strains.

Original Δ*hp0135* Mutant (Strain RIH3)					
**NCBI Gene** **Designation**	***H. pylori* 26695** **Locus Tag**	**Description**	**^a^ Mutation**	**^b^ Impact**	**^c^ Frequency**
*fliP*	HP0684/HP0685	flagellar export apparatus protein FliP	(C)_8→7_ (261/747 nt)	Pro87fs	98.8%
*CV725_RS06500*	HP0499	phospholipase A (PldA), pseudogene	(G)_10→9_ (683/1070 nt)	remains phase ‘off’	97.5%
*CV725_RS07900*	HP1572	lytic transglycosylase MltD	codon-207 (TAT→CAT)	Tyr207His	100%
*CV725_RS08585*	HP0135	small lipoprotein	Δ73 bp (Δ35-107/135 nt)	deletion	100%
motile variant of Δ*hp0135* (strain RIH3A)					
*CV725_RS08295*		hypothetical protein, pseudogene	(C)_14→13_ (74/351 nt)	phase ‘off’ to phase ‘on’	88.8%
*fur*	HP1027	ferric iron uptake transcriptional regulator (Fur)	(A)_8→9_ (55/453 nt)	Asn21fs	96.5%
*CV725_RS06500*	HP0499	phospholipase A (PldA), pseudogene	(G)_10→8_ (683/1070 nt)	phase ‘off’ to phase ‘on’	89.9%
*CV725_RS07900*	HP1572	lytic transglycosylase MltD	codon-207 (TAT→CAT)	Tyr207His	99.2%
*CV7255_RS08585*	HP0135	small lipoprotein	Δ73 bp (Δ35-107/135 nt)	deletion	100%
motile variant of Δ*hp0135* (strain RIH3C)					
*CV725_RS08295*		hypothetical protein, pseudogene	(C)_14→13_ (74/351 nt)	phase ‘off’ to phase ‘on’	88.8%
*fur*	HP1027	ferric iron uptake transcriptional regulator (Fur)	codon-140 (GTG→ATG)	Val140Met	99.6%
*CV725_RS06500*	HP0499	phospholipase A (PldA), pseudogene	(G)_10→8_ (683/1070 nt)	phase ‘off’ to phase ‘on’	84.9%
*CV725_RS07900*	HP1572	lytic transglycosylase MltD	codon-207 (TAT→CAT)	Tyr207His	100%
*CV7255_RS08585*	HP0135	small lipoprotein	Δ73 bp (Δ35-107/135 nt)	deletion	100%

^a^ Mutations were identified by comparing the whole genome sequences with the published NCBI genome for *H. pylori* B128 (Accession no.: NZ_CP024951.1). Silent mutations that changed a codon but did not alter the amino acid residue and mutations in intergenic regions are not shown. ^b^ Indicates how the mutation impacts the coding sequence—‘fs’ designates a frameshift in the coding sequence at the indicated amino acid position. ^c^ Indicates the frequency at which the mutation was observed in reads for that region. A frequency of 80% was used as the threshold for calling mutations.

## Data Availability

The original contributions presented in the study are included in the article and Supplementary Materials; further inquiries can be directed to the corresponding author.

## Supplementary Materials

The following supporting information can be downloaded at: https://www.mdpi.com/article/10.3390/molecules30020204/s1, Table S1: Proteins identified from the flagellar preparation; Table S2: Proteins identified in the Sarkosyl-insoluble fraction; Table S3: Proteins identified from the Sarkosyl-soluble fraction; Table S4: Proteins identified from the murein sacculus preparation.
